# Phosphosulindac (OXT-328) restores the suppressed corneal sensitivity in rabbits with dry eye disease: therapeutic implications

**DOI:** 10.3389/fddev.2026.1778440

**Published:** 2026-06-03

**Authors:** Wei Huang, Ziyi Wen, Konstantinos Tourmouzis, Liqun Huang, Sanford M. Goldstein, Ernest Natke, Robert Honkanen, Basil Rigas

**Affiliations:** 1 Department of Ophthalmology, Stony Brook University, Stony Brook, NY, United States; 2 Department of Medicine, Stony Brook University, Stony Brook, NY, United States; 3 Department of Cornea and Ocular Surface, Changsha Aier Eye Hospital, Changsha, Hunan, China; 4 Barts and the London School of Medicine and Dentistry, London, United Kingdom; 5 Medicon Pharmaceuticals, Inc., Setauket, NY, United States; 6 Apis Therapeutics LLC, Setauket, NY, United States; 7 Department of Preventive Medicine, Stony Brook University, Stony Brook, NY, United States

**Keywords:** corneal sensitivity, drug formulations, dry eye disease, cyclosporine, ocular analgesia, ocular surface, phosphosulindac

## Abstract

**Background:**

The symptoms of dry eye disease (DED) result from activation of ocular sensory nerves and constitute the dominant component of its clinical presentation. We assessed the effect of phosphosulindac (PS), a small molecule efficacious in the treatment of DED in preclinical models, on corneal sensitivity (CS).

**Methods:**

CS was determined with a Cochet-Bonnet esthesiometer in New Zealand white (NZW) and Dutch-belted black (DBB) rabbits. DED was induced by Concanavalin A injections into the rabbits’ lacrimal glands. Changes in CS, tear osmolarity, tear break up time (TBUT) and Schirmer tear test were measured before and after DED induction. PS in various formulations (emulsions, nanoparticles and hydrogels) and other ocular drugs were applied in eye drop form to normal rabbits and those with DED.

**Results:**

nduction of DED caused a decrease in the CS, TBUT time as measured by fluorescein dye, in tear production as measured by Schirmer’s tear test and an increase in tear osmolarity. PS markedly restored the suppressed CS in dry eyes. The effect was immediate, fully reversible, lasted ∼26 h and appeared dissociated from its anti-inflammatory properties. The most efficacious formulation was a Carbopol-based hydrogel; cyclodextrin-based and emulsion formulations were also effective. The most optimal dose of PS was 0.2% and the optimal pH was 6.2. None of 7 compounds structurally related to PS affected CS nor did cyclosporine, lifitegrast and artificial tears. PS does not have an anesthetic effect on the cornea. In normal eyes, PS suppressed CS and this effect was concentration-, formulation-, and pH-dependent. Two non-steroidal anti-inflammatory drugs (NSAIDs), ketorolac and bromfenac, and lidocaine suppressed CS in normal but not in dry eyes.

**Conclusion:**

PS restores the suppressed CS in dry eyes possiblely by a direct effect on corneal nerves. This effect appears unique to PS, distinct from all tested compounds including the two currently approved drugs for DED. PS, in addition to affecting CS of DED, may improve its symptoms and merits further evaluation for the treatment of DED.

## 1 Introduction

Dry eye disease (DED), a prevalent multifactorial disease of the ocular surface characterized by a loss of homeostasis of the tear film accompanied by ocular symptoms, in which tear film instability and hyperosmolarity, ocular surface inflammation and damage, and neurosensory abnormalities play etiological roles (Craig et al., 2017; Paulsen et al., 2014; Vehof et al., 2014; Tan et al., 2015; Smith et al., 2007; Mondal et al., 2023; Aragona et al., 2025; Clayton, 2018; Yang et al., 2026). The etiology of DED is traditionally classified into aqueous-deficient dry eye, involving reduced tear production often linked to Sjögren syndrome, and evaporative dry eye, which is frequently caused by meibomian gland dysfunction (MGD) (Barabino et al., 2025). Pathophysiologically, hyperosmolar stress on the ocular surface epithelium activates intracellular signaling pathways, such as MAPK and NF-κB, leading to the release of pro-inflammatory cytokines like TNF-α, IL-1β, and IL-6 (Barabino et al., 2025). Recent research also highlights the role of “dysregulated parainflammation,” an intermediate immune response that, when persistent, transitions into chronic inflammation and contributes to goblet cell loss and neurosensory abnormalities.

Topical cyclosporine A and lifitegrast remain the gold standard for DED. Given, however, recent advances in the understanding of DED pathophysiology, its therapeutic landscape has shifted toward targeted, water-free delivery systems and neurostimulation. Perfluorohexyloctane (Miebo) (approved in 2023) reduces tear evaporation by forming a semi-fluorinated protective layer (Alija et al., 2026; Bacharach et al., 2025), while Lotilaner (Xdemvy) addresses Demodex-associated inflammation, a previously under-recognized contributor to DED (Fabara et al., 2025). In patients with aqueous deficiency, Acoltremon (Tryptyr) (approved in 2025) stimulates endogenous tear production via corneal nerve activation (Moshfeghi, 2026; Pattar et al., 2026). These pharmacologic approaches are increasingly complemented by in-office radiofrequency therapy, which restores meibomian gland function and offers a durable alternative to chronic topical regimens.

Ocular pain, often accompanied by light sensitivity, foreign-body sensation, dryness, and irritation, is frequent (Clayton, 2018). The most consistent clinical feature of DED is what is described as chronic dry eye-like pain (Rosenthal and Borsook, 2016). Many patients with mild to moderate DED describe their symptoms as irritating rather than painful, while others describe features of neuropathic pain, sometimes presenting without signs of epithelial damage (McMonnies, 2017).

The diagnosis of DED is complicated by an inconsistent correlation between reported symptoms and observed signs, reflecting inherent limitations of current clinical tests, the variability of the disease and pain thresholds (Clayton, 2018; Ong et al., 2018). DED may be predominantly symptomatic but without signs of ocular surface disease or the reverse (Clayton, 2018; McMonnies, 2017).

DED can involve symptoms that are both nociceptive (the physiological response to noxious stimuli) and neuropathic (damaged somatosensory nerves). The latter may explain the dichotomy between signs and symptoms in some DED patients or DED symptoms associated with reduced corneal sensitivity. The free corneal nerve endings between the superficial epithelial cells, very near to the ocular surface, are damaged from, for example, tear evaporation and ocular surface inflammation (Galor et al., 2015).

The sensory nerve function is evaluated by measuring ocular surface sensitivity. Esthesiometry, the measurement of tactile sensation, has been used extensively to study the origin of DED symptoms and the contribution of the ocular surface nerves to the clinical picture. The Cochet-Bonnet esthesiometer, evolved from the one first described in 1894 (Martin and Safran, 1988), has been used extensively to determine ocular surface sensitivity. Several studies have measured corneal and conjunctival sensitivity in subgroups of DED patients, pursuing correlations between esthesiometry results and ocular symptoms (Bourcier et al., 2005; Situ et al., 2008; Belmonte et al., 2017; Adatia et al., 2004; Dien et al., 2015).

Given the lack of an optimal treatment for DED, we recently explored the efficacy of phospho-sulindac (PS, OXT-328), a novel anti-inflammatory drug (Huan et al., 2011; Mattheolabakis et al., 2013), in the treatment of DED. Using an improved rabbit model of chronic DED, we demonstrated the remarkable efficacy of PS (Honkanen et al., 2018). In particular, PS, applied topically as eye drops, restored to normal the tear break-up time, tear osmolarity, and tear lactoferrin levels. PS showed no side effects and was much more efficacious than cyclosporine or lifitegrast.

Since ocular pain in its various manifestations is an important, if not the main, component of the symptoms of DED, we assessed the effect of PS on CS in dry and normal rabbit eyes and compared it to that of structurally similar compounds. We also measured the effect of ophthalmic drugs on CS in normal eyes and dry eyes. Some such as local anesthetics (proparacaine and benoxinate) are used to numb the ocular surface to facilitate diagnostic testing such as the Schirmer Tear Test, measurements of intraocular pressure or assessment of corneal damage. Another local anesthetic, lidocaine, is used to provide immediate and temporary relief from severe ocular pain, burning and irritation especially during procedures. For contrast, we have also tested the effect of two NSAIDs on CS in normal and dry eye. Both are anti-inflammatory agents which are used as either short-term therapies or are used to treat post-operative inflammation. We also tested the response to artificial tears that are used to manage mild to moderate dry eye. Finally, we tested two dry eye therapies, cyclosporine and lifitegrast, which have known immunomodulatory actions, which have longer onset times but are used to treat patients who have not found relief from over-the-counter remedies.

Here, we report our findings demonstrating the ability of PS in various formulations to normalize the suppressed CS in dry eyes while, in contrast, PS suppresses CS in normal eyes.

## 2 Materials and methods

### 2.1 Reagents and study drugs

PS was a gift from Medicon Pharmaceuticals, Inc (Setauket, NY). Concanavalin A (Con A), fluorescein, lidocaine, and most routine chemicals were purchased from Sigma, St. Louis, MO. Ophthalmic solutions of ketorolac trometamol (0.5%), bromfenac (0.09%), cyclosporine (0.05%, Restasis®), lifitegrast (5%, Xiidra®), proparacaine (0.5%, Sandoz®), lidocaine (3.5%), fluorescein sodium/benoxinate hydrochloride (0.25%/0.4%, Altefluor Benox®), and one brand of artificial tears (0.5% carboxymethylcellulose, Refresh Plus®) were used as commercially available.

### 2.2 Drug formulation

The various formulations of PS used in these studies are summarized in Table 1, including their identifiers (F1–F9). An overview of formulation classes and key characteristics is provided in Table 1, while detailed compositions are presented in Supplementary Table S1. The preparation of these formulations is briefly described below.

**TABLE 1:** Overview of phosphosulindac (PS) ocular formulations and key characteristics.

Formulation	Type	PS (%)	Key system/Design feature	pH range
F1	Solution	3.5	High cyclodextrin (HP-β-CD)	4.0
F2	Solution	1.0	Moderate cyclodextrin	4.0
F3	Solution	0.5	Cyclodextrin-based; pH optimization	4.0–8.0
F4	Solution	0.05–1.6	Multi-excipient system; dose-response	7.4
F5	Solution	3.5	Vitamin E TPGS-based solubilization	6.7
F6	Emulsion	1.0–2.0	Oil-in-water emulsion	6.1
F7	Nanoparticle	3.5	Polymer-based nanoparticles (mPEG-PLA)	7.1
F8	Hydrogel	0.2–0.6	Carbopol-based sustained-release gel	6.4
F9	*In situ* gel	3.0	Gellan gum-based *in situ* gel	6.7

Abbreviations: HP-β-CD, hydroxypropyl-β-cyclodextrin; TPGS, D-α-tocopheryl polyethylene glycol succinate; mPEG-PLA, methoxy poly (ethylene glycol)-poly (lactide).

Full composition details are provided in Supplementary Table S1.

#### 2.2.1 Solution formulations

To prepare the solution formulations F1-F3 (2-hydroxypropyl)-β-cyclodextrin (HP-β-CD) and Tween 80 were dissolved in water. PS was added into the above solution and stirred at 50 °C until PS was fully dissolved. The pH was adjusted to the required value. To prepare the *solution formulation* F4, first, polyvinyl alcohol was dissolved into water by stirring at 95 °C for 6 h and all other ingredients shown in Table 1, including PS, were added into a glass vial, stirred at 50 °C for 4 h and then stirred at RT overnight. The pH was adjusted to 7.4 ± 0.2 using NaOH and the osmolality to 280–320 mOsm/kg H_2_O. To prepare the F5 formulation, polyquaternium-1 and Vitamin E TPGS (D-α-Tocopheryl polyethylene glycol 1,000 succinate) were dissolved in purified water, PS was added to this solution, and stirred at 70 °C for 30 min. This solution was then centrifuged at 13,200 rpm for 10 min and the supernatant was collected. Mannitol and boric acid were added to the harvested supernatant. After pH adjustment to 6.7 ± 0.2 using NaOH, purified water was added to the final volume. All final solutions were sterilized by filtration through a 0.22 µm syringe filter.

#### 2.2.2 Emulsion formulation

To prepare the emulsion formulation (F6), PS was dissolved in propylene glycol by stirring at 50 °C. This was followed by the addition of mineral oil and stirring to prepare the oil phase. HP-β-CD, Kolliphor EL and Tween 80 were dissolved into water to prepare the water phase, which was then added into the oil phase. The system was emulsified by probe sonication (Branson 150, Fisher Scientific™) 8 times, each for 5 s, with 5 s intervals between them. The resulting emulsion was sterilized by filtration through a 0.22 µm film.

#### 2.2.3 Nanoparticle formulation

For the nanoparticle (NP) formulation of PS (F7), NPs were prepared by the emulsion-evaporation method as previously described (Honkanen et al., 2018). Briefly, the oil phase was obtained by dissolving PS and mPEG-PLA (methoxy poly (ethylene glycol)-poly (lactide)) polymer in dichloromethane. The water phase was prepared by dissolving 365 mg of sodium cholate in 60 mL of purified water. Five mL of the oil phase were gently added into 15 mL of the water phase. The emulsion was prepared by probe sonication (2 min at 75% output; Branson 150, Fisher Scientific™). Then the dichloromethane was fully evaporated by stirring overnight. The NP solution was concentrated and washed with PBS 3 times using ultrafilter tubes (Amicon Ultra-15, 10 kDa MWCO, Millipore Sigma). The concentrated NPs were resuspended into PBS to a final volume of 5 mL, transferred to an Eppendorf tube and spun for 7 s to remove any aggregates. The supernatant was harvested as the final product.

#### 2.2.4 Hydrogel formulations

To prepare the *carbopol-based* hydrogel formulation (F8), carbopol 980 was dissolved into water to a concentration of 0.9% and the pH was adjusted above 7.0 to form a gel. A stock solution of PS 1.2% or 0.4% was prepared and 1 mL of it was mixed with 1 mL of 0.9% Carbopol gel, and vortexed to make the PS carbopol gel. To prepare the *gellan gum-based* hydrogel (F9), first, a gellan gum solution was prepared by adding gellan gum to deionized water and heating the mixture to 90 °C with fast stirring (500 rpm). Once the gellan gum was completely dissolved, the solution was sterilized by filtration through a 0.22 µm syringe filter. Then, PS and other excipients (Vitamin E TPGS and HP- β-CD) were added to the system and stirred at 50 °C at 500 rpm until complete dissolution.

### 2.3 Animal studies

All animal studies were approved by the IACUC of Stony Brook University and followed the guidelines of the Association for Research in Vision and Ophthalmology (ARVO) Statement for the Use of Animals in Ophthalmic and Vision Research.

We used male rabbits, mainly NZW rabbits (Charles River Laboratories, Kingston NY) and in some studies DBB rabbits (Convance Inc. New York, NY) were used, about 10 months of age without ocular diseases. Rabbits were singly housed in rooms where the temperature (21°F ± 2.5°F) and humidity (40% ± 5%) were strictly controlled and had unlimited access to water and standard rabbit chow. Rabbits were acclimated for at least 2 weeks after their arrival, and before their nictitating membranes were removed.

### 2.4 Induction of dry eye

The induction and assessment of dry eye in NZW rabbits was recently described by us (Honkanen et al., 2018). Briefly, 2 weeks after removal of the nictitating membranes, we obtained the baseline values of tear osmolarity, tear breakup time (TBUT), and Schirmer tear test (STT). To induce dry eye, the next day we injected Con A (Con A dissolved in PBS) into all lacrimal glands of the rabbits under ultrasound guidance and with the rabbits under deeper anesthesia with isoflurane. This included the palpebral (1,000 µg Con A, 0.2 mL) and the orbital (500 µg Con A, 0.1 mL) portions of the superior lacrimal gland, and the inferior lacrimal gland (1,000 µg Con A, 0.2 mL) of each eye. The success of the injection into the inferior lacrimal gland was confirmed by ultrasonography. Repeat assessment of tear osmolarity, TBUT, and STT were performed on day 5 after ConA injections, confirming the induction of dry eye. The methodology for the determination of each one of these parameters has been detailed elsewhere (Honkanen et al., 2018).

### 2.5 Treatment with phospho-sulindac (PS) and other ocular drugs

On day 5 or 6 following induction of Dry Eye, rabbits were given different formulations of PS and other test drugs. Following administration of these test substances, repeated measures of CS were performed by Cochet-Bonnet esthesiometry. In normal animals, the timeline was similar in that after the 2 weeks recovery from the removal of the nictating membranes, PS and other ocular drugs were not administered until an additional 5 or 6 days had passed. As in experiments with dry eye animals, drug administration was followed by repeated measures of corneal sensitivity determined by Cochet-Bonnet esthesiometry.

### 2.6 Corneal touch threshold (CTT) determination

CS was measured with the Cochet-Bonnet esthesiometer (Luneau, France) in a quiet examination room as previously described (Dorbandt et al., 2017; Cantarella et al., 2017). The esthesiometer contains a nylon filament 0.1 mm in diameter; its maximal length used in testing is 6 cm. All measurements were performed by the same investigator (WH), around the same time of day, and in the same room to minimize the effect of diurnal and environmental variability. Animals were placed in the same device and positioned identically for every session. The investigator was blinded with respect to which test substance was administered.

Briefly, CTT testing started with the nylon filament applied perpendicularly to the central cornea (its most sensitive area) at its full length of 6 cm. If a positive response (full blink and retraction of the eye into the ocular orbit) was not noted on 3-5 attempts, the filament was incrementally retracted (shortened) in 5 mm steps until a stimulus evoked a response. The length of filament simulating a positive response was then recorded.

The baseline value (before treatment with the test drug) was recorded as 0 min. After a single 25-µL eye drop of PS was applied onto the ocular surface, esthesiometry measurements were performed every 5 min for the first 50 min and then every 10 min for the next 50 min or longer time. Each CS study was performed on at least 4 eyes (4 rabbits).

### 2.7 Statistical analysis

Average AUC levels were compared between groups using one-way analysis of variance (ANOVA) models followed by pairwise *post hoc* comparisons to determine the nature of significant effects. All tests were two-sided and considered significant at the α = 0.05 threshold. A paired t-test was used to compare the CS before and after drug administration as noted in the legend for Figure 2A.

## 3 Results

### 3.1 The effect of PS on CS in dry eyes

CS is significantly altered in patients with dry eye, the result of ocular surface inflammation and/or damage to nerve endings (McMonnies, 2017). We, therefore, studied the effect of PS and other drugs on CS in rabbits with dry eye induced by injecting ConA into their lacrimal glands (Honkanen et al., 2018). The induction of dry eye was confirmed by the expected changes in three functional parameters on day 5 post ConA injection. Specifically, compared to baseline, TBUT was decreased by 80.7% (baseline = 60 ± 0.0 s vs. day 5 = 11.6 ± 3.4 s; *mean ± SEM* for this and subsequent values; p < 0.0001); STT was decreased by 51.4% (baseline = 15.5 ± 1.9 mm vs. day 5 = 7.5 ± 1.0 mm; p < 0.001), and tear osmolarity was increased by 4.7% (baseline = 303.6 ± 3.7 mOsm/L vs. day 5 = 317.9 ± 2.7 mOsm/L; p < 0.0001).

As expected, rabbits with dry eyes required significantly more force to elicit a blink response as determined by the much shorter filament length as measured using the Cochet-Bonnet esthesiometer compared to normal eyes (filament lengths of dry eye rabbits = 3.8 ± 0.1 cm vs. normal control rabbits = 5.7 ± 0.1 cm; p < 0.001). This is analogous to the human condition. Thus, animals with dry eyes show a decreased corneal sensitivity when compared to normal control animals.

**FIGURE 1:**
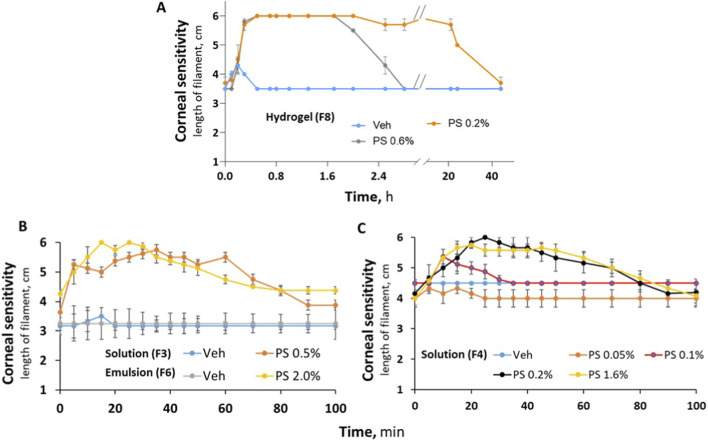
Formulation and dose effects on the efficacy of PS in CS in rabbits with dry eye disease. CS was determined by a Cochet-Bonnet esthesiometer in NZW rabbits with Concanavalin A-induced dry eyes. PS formulations are detailed in Table 1. **(A)** The two different concentrations of PS in the F8 hydrogel formulation significantly restored CS in dry eyes (p < 0.02 for both), and that effect of 0.2% PS much longer than 0.6% PS. **(B)** PS in a cyclodextrin-based (F3; pH 6.0) or an emulsion formulation (F6) significantly restored CS in dry eyes (p < 0.03 for both). **(C)** The two highest of four different concentrations of PS in the F4 solution formulation significantly restored CS in dry eyes (p < 0.05 for both) but the difference between them was not statistically significant (p > 0.4), and the lowest of four different concentrations of PS had no effect (p > 0.1). Each eye received drug dissolved in 25–50 µL of solution. Values: *mean ± SEM*; n = 4 eyes/time point.

To test the effect of PS on animals with dry eyes, we used eye drops of four different formulations (F3, F4, F6 and F8) of PS as described in Table 1. PS was most efficacious in the F8 hydrogel formulation (Figure 1A). A single eye drop of PS 0.2% in this formulation, applied topically to the ocular surface of NZW rabbits with dry eyes restored to normal their (suppressed) CS when compared to vehicle, which only had a small short-lived effect (p < 0.02). The effect of PS was essentially immediate, becoming apparent at 5 min, the first time point tested after drug application, and lasted for 27 h, before it started dissipating. This effect had an unusual dose depenence, in that the effect of a higher concentration of PS (0.6%) was of shorter duration (2.5 h).

Both the solution (F3, F4) and emulsion (F6) formulations restored to normal the corneal sensitivity of rabbits with dry eyes (Figures 1B,C), but their effect, roughly similar, was of shorter duration, with the normalization of CS lasting less than 1 h. As shown in Figure 1B, the F3 and F6 formulations of PS significantly improved CS (p < 0.03 for both); their respective ACU_0–90 min_ values were 132.2 ± 4.5 cm*min and 75.4 ± 18.0 cm*min. It merits pointing out that all efficacious formulations had a similarly rapid effect that peaked 20 min after PS’s application to the eye.

In particular, the F4 formulation, studied in greater detail (Figure 1C), produced an effect that was dose-dependent. While the lowest dose of PS (0.05%) had no effect on CS (p > 0.1), the effect of higher doses of 0.2% and 1.6% was evident. Interestingly, even though these two concentrations, 0.2% and 1.6%, differ by 8-fold from each other, they produced a quantitatively similar effect (AUC_0–90 min_ of 90.4 ± 19.7 cm*min and 112.7 ± 13.0 cm*min; p > 0.4), indicating a sharp transition in the dose response relationship between PS and CS.

### 3.2 The effect of local anesthetics, NSAIDs, and dry eye therapeutics on CS in dry eyes

We evaluated the effect on CS of four groups of compounds clinically used in ophthalmology: the topical anesthetics (lidocaine, proparacaine and benoxinate) (Page and Fraunfelder, 2009; Kumar et al., 2015; Schlegel and Swan, 1954); two NSAIDs (ketorolac and bromfenac) widely-used as ocular anti-inflammatory and analgesic drugs (Wilson et al., 2015; Ahuja et al., 2008); the two approved drugs for DED (cyclosporin and lifitegrast) (Thulasi and Djalilian, 2017); and artificial tears (containing 0.5% carboxymethylcellulose as its active ingredient) that provide relief from ocular surface irritation and pain associated with DED (Moshirfar et al., 2014).

**FIGURE 2:**
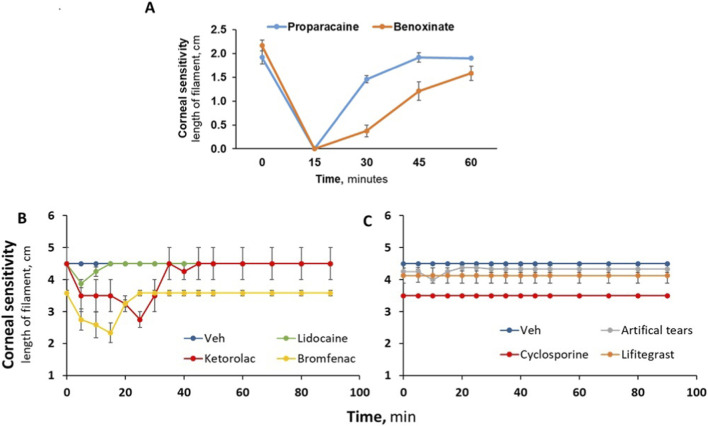
The effect of topical anesthetics, NSAIDs and DED therapeutics on CS in rabbits with dry eyes. CS was determined by a Cochet-Bonner esthesiometer in NZW rabbits with Concanavalin A-induced dry eyes. **(A)** The two topical anesthetics (proparacaine and benoxinate) decreased CS in dry eyes. Using a paired t-test, the baseline CS value at T0 (before drug administration) was compared to the CS value at T15 (after drug administration) for each (p < 0.0001 for both). **(B)** Bromfenac and ketorolac both decreased CS (p < 0.05 for both) in dry eyes animals, while lidocaine did not (p > 0.05). **(C)** Artificial tears, cyclosporine and lifitegrast had no effect on CS in rabbits with dry eye (p > 0.05 for all). Vehicle was normal saline (all compounds except cyclosporine are water soluble; no vehicle was required for cyclosporine as it lacked any efficacy). The volume of eye drops were 25–50 µL. Values: *mean ± SEM*; n = 4 eyes/time point.

As shown in Figure 2A, topical anesthetics, proparacaine and benoxinate, further decreased CS in rabbits with dry eyes to levels unmeasureable by the Cochet-Bonner esthesiometer almost immediately after their administration. CS remained depressed for 15 min before CS began to improve and return to baseline by 45–60 min post administration. In similar studies, application of the NSAIDS, bromfenac and ketorolac, also further decreased CS in dry eye animals for up to 15 min and 25 min, respectively. The effect of lidocaine was much more transient (Figure 2B) where as vehicle had no effect. Other ophthalmic drugs approved for treatment of DED, cyclosporine and liftegrast, had no detectable effect on the decrease of CS in this model. Likewise, the application of artificial tears had only a transient effect on CS (Figure 2C).

### 3.3 The effect of PS on CS in normal eyes

**FIGURE 3:**
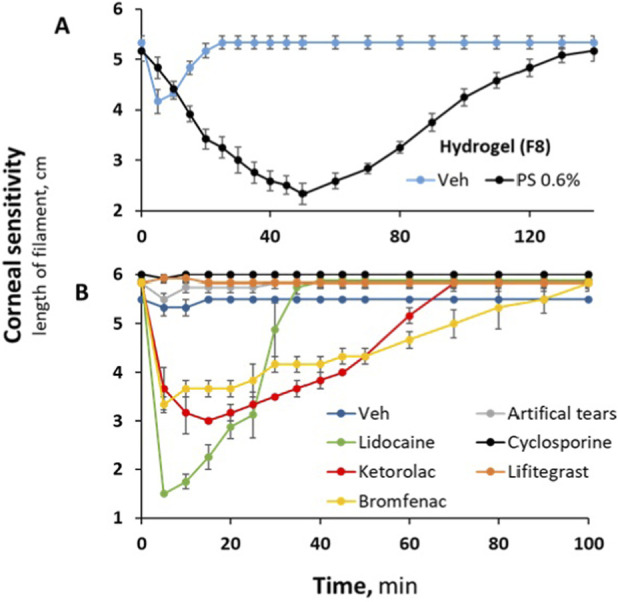
The effect of PS and lidocaine, NSAIDs and DED therapeutics on CS in rabbits with normal eyes. CS was determined using a Cochet-Bonner esthesiometer. **(A)** PS in a hydrogel formulation (F8) suppresses CS compared to vehicle (p < 0.0004). **(B)** Lidocaine, bromfenac and ketorolac suppressed CS when compared to vehicle. Where as Cyclosporine, Lifitegrast and artificial tears had no effect on CS. Vehicle was normal saline (all compounds except cyclosporine are water soluble; no vehicle was required for cyclosporine as it lacked any efficacy). The administer volume of eye drops was 25–50 µL. Values: *mean ± SEM*; n = 4 eyes/time point.

We evaluated the effect of PS on normal rabbit eyes. As shown in Figure 3A, one drop of topically applied PS in hydrogel significantly suppressed the CS of normal NZW rabbits when compared to vehicle. The effect was quantitatively pronounced and rapid. It became apparent at 5 min and the peak reduction of 55.8% occurred at 50 min, when a rather slow decay began, returning CS to baseline at 140 min. In contrast to PS, the vehicle had a very brief (25 min) and weak (21.9% maximal reduction) effect that was not statistically significant.

As compared to dry eyes, we evaluated the effect on CS of lidocaine, cyclosporin, lifitegrast, the same brand of artificial tears, and the ocular NSAIDs ketorolac and bromfenac on normal eyes. Figure 3B shows that, in keeping with its known anesthetic effect (Page and Fraunfelder, 2009), lidocaine decreased CS rapidly and significantly (p < 0.001) for about 30 min. When compared the peak effect of lidocaine was stronger than that of PS (74.4% vs. 55.8%) but significantly shorter in duration (30 min vs. 120 min). The AUC values make their difference between lidocaine and PS more apparent (AUC_0–90 min_ of 95.0 ± 4.9 cm*min verses 202.1 ± 22.8 cm*min; p < 0.05, respectively). Much like their lack of an effect in rabbits with dry eyes, cyclosporine, lifitegrast and the artificial tears had no effect on CS. Ketorolac and bromfenac which suppressed CS in animals with dry eyes also had the same effect on CS in animals with normal eyes. The potency of the two ocular NSAIDS on CS was equivalent (Figure 3B; p < 0.004). Their effect was similar to that of 0.2% PS in liquid F4 formulation (see below).

### 3.4 The formulation, concentration, and pH dependence of the effect of PS on normal eyes

We studied several concentrations of PS in various formulations and in one of which we varied the pH. Our results establish that formulation, dose and pH affect the CS response of normal eyes to PS.

**FIGURE 4:**
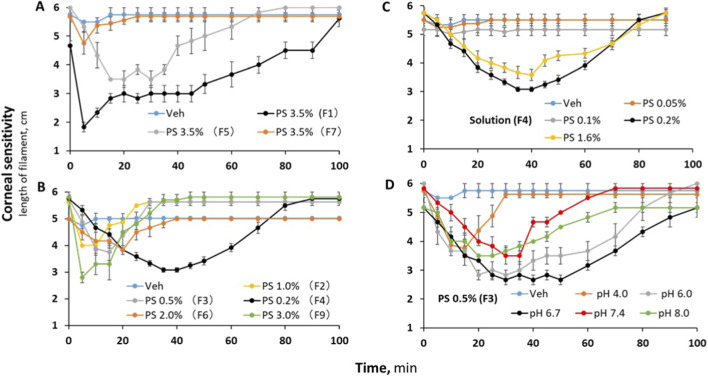
Formulation, dose, and pH effects on the efficacy of PS in CS with normal eyes. CS was determined in NZW rabbits using a Cochet-Bonner esthesiometer. **(A)** Effect of the same concentration of PS dissolved in three different formulations (F1,F5 and F7) on CS. The effect of PS in F1 and F5 had a significant effect on CS (p < 0.04 for both), but not as formulated in F7. **(B)** PS formulated in an *in situ* gel (F9) or in solution (F2, 3) or emulsion (F6) formulations generated varied results. The effect of PS in F2, F6 and F9 was significant (p < 0.04 for all), but was restricted to the first 40 min. The effect of PS in F3 was not significant (p > 0.06). PS as formulated in F4 decreased CS to a greater extent and for a longer period of time. **(C)** Dose response of 4 concentrations of PS in F4 solution formulation. The effect of the two lowest concentrations (PS 0.05% and PS 0.1%) was not significant (P > 0.5) when compared to vehicle, while that of the higher two (PS 0.2% and PS 1.6%) was compared to vehicle (p < 0.0002 for both); while the effect of the higher two concentration was the similar (p > 0.5). Values: mean ± SEM; n = 4 eyes/time point. **(D)** Effect of pH on PS 0.5% in the F3 cyclodextrin-based formulation on CS. The decrease in CS was greatest at pH is 6.2. Values: *mean ± SEM*; n = 4 eyes/time point. The volume of eye drops was 25–50 µL.

As expected (Morrison and Khutoryanskiy, 2014), how the PS solution was formulated had a major effect on its efficacy. All three formulations (Figure 4A), F1, F5 and F7, had the same concentration of PS (3.5%), but exhibited markedly different results on CS. The one with a high concentration of HP-β-CD (F1) was most efficacious (AUC = 115.0 ± 22.1 cm*min, p < 0.04); the formulation based on Vitamin E TPGS (F5) gave an intermediate result (AUC = 99.2 ± 16.7 cm*min, p < 0.03); and the nanoparticle formulation (F7) had no effect (AUC = 9.4 ± 4.3 cm*min, p > 0.1).

Figure 4B shows the varying responses of CS to PS formulated in five additional formulations (solution, emulsion and *in situ* gel). Of them, the cyclodextrin-based formulations (F1-F3; Figures 3A,B) significantly suppressed CS at PS concentrations of 3.5% and 1.0% (p < 0.04 and p < 0.02); notably, cyclodextrin can accommodate significant amounts of PS in its hydrophobic pocket (Jansook et al., 2010). A lower PS concentration of 0.5% dissolved in cyclodextrin (F3) was ineffective (p > 0.06), producing only a mild and brief (20 min) suppression of corneal sensation. The emulsion formulation (F6) and ocular gel formulation based on gellan gum (F9), showed a significant effect (p < 0.04 for both), which, surprisingly, dissipated by 40 min, in contrast to expectations for a more prolonged effect based on a longer dwell time of the drug on the ocular surface (Destruel et al., 2017).

To examine the dose response relationship between PS and CS in normal eyes (Figure 4C), we varied the concentration of PS from 0.05% to 1.6% in the same formulation of F4. The two lowest concentrations, 0.05% and 0.1%, had essentially no effect on CS, being similar to control vehicle (p > 0.5 for both vs. control vehicle). Both PS 0.2% and 1.6% were efficacious (p < 0.0002 vs. control vehicle). Interestingly, the effect of PS 0.2% was similar with that of 1.6%; their respective ACU_0–90 min_ are 135 ± 8.0 cm*min and 107.1 ± 10.4 cm*min (p > 0.5 compared to each other). The latter is similar to the duration of the effect of PS in dry eyes, where PS 0.6% had a shorter effect on CS that 0.2% (Figure 1A). Another interesting aspect of the dose dependence of PS’s effect is the sharp transition from ineffective to a significant effect. For example, in Figure 1C, PS 0.05% and 0.1% have no effect on CS, contrasting to the significant effect of 0.2% and 1.6%; the difference in efficacy between PS 0.1% and PS 0.2% is striking (p < 0.0001 compared to each other).

Figure 4D shows that the effect of PS on CS in normal eyes is dependent on the pH of the formulation. PS 0.5% in the F3 cyclodextrin formulation ranged in efficacy from a very modest and brief effect at pH 4 (p > 0.06) to its strongest efficacy at pH 6.0 (p < 0.007). The pH optimum for this effect is 6.2. Formulations of PS with pH values above or below this pH were less efficacious.

### 3.5 The structural specificity of the effect of PS on normal rabbit eyes

**FIGURE 5:**
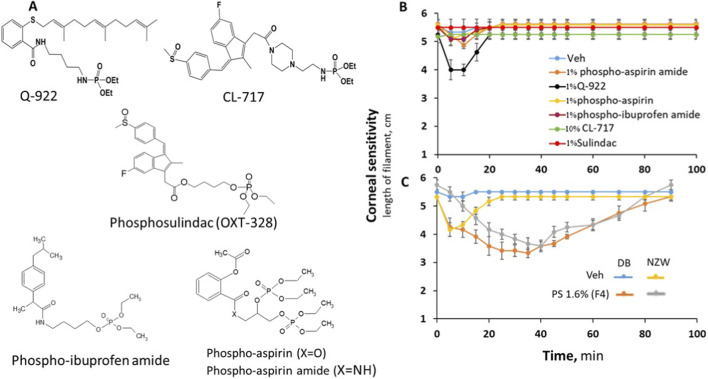
The effect of PS related compounds on CS in rabbits with normal eyes. CS was determined a Cochet-Bonner esthesiometer. **(A)** Shows the structures of PS, two derivatives of sulindac (Q-922 and CL-717) as well as phospho-ibuprofen amide, phosphor-aspirin and its amide. **(B)** The effect of 6 structurally related compounds on CS. Only drug Q-922 had a significant effect (p < 0.003) to PS (not shown), but it was mild and brief. **(C)** The effect of rabbit species on the efficacy of PS on CS with normal eyes. There was no significant difference between the efficacy of PS in decreasing CS in NZW rabbits and DBB rabbits (p > 0.7). Values: *mean ± SEM*; n = 4 eyes/time point. The volume of the eye drops were 25–50 µL per application.

We studied six compounds sharing structural similarities to PS (Figures 5A,B). They include: sulindac, a structural component of PS; two derivatives of sulindac (CL-717 and Q-922); a derivative of the NSAID ibuprofen; and two derivatives of aspirin differing in their linkage to the spacer moiety (carboxylic ester vs. amide bond). In contrast to PS, all failed to have any appreciable effect on CS in rabbits with normal eyes, with the exception of Q-922 whose effect was significant (p < 0.003) but mild and brief.

### 3.6 Comparative evaluation of PS in albino and pigmented rabbits

NZW rabbits are albinos, a feature that makes them attractive animal models for ocular studies (Esteves et al., 2018; Zernii et al., 2016). On the other hand, some drugs bind to melanin in pigmented ocular tissues. Such binding may lead to drug accumulation in the pigmented tissues and prolonged drug retention, affecting ocular drug delivery and biodistribution and ultimately their efficacy (Rimpela et al., 2018). Thus, we also studied the effect of PS on CS in DBB rabbits, known to have adequate melanin levels in ocular tissues (Durairaj et al., 2012). As shown in Figure 5C, PS 1.6% suppressed CS in DBB rabbits to a similar extent as in NZW rabbits, their AUCs being essentially identical (AUC_0–90 min_ of 107.1 ± 10.4 cm*min and 102.3 ± 12.0 cm*min; p < 0.0003 for each as compared to vehicle control). In this study, the vehicle control seemed to have a much greater effect (albeit of short duration) on CS in NZW rabbits than in the pigmented DBB rabbits.

## 4 Discussion

Our results demonstrate a significant effect of PS on CS in the eyes of the rabbit, which was remarkable for the prompt restoration of the suppressed CS of dry eyes and for the opposite effect on normal eyes. Each of these antithetic effects is discussed below.

### 4.1 Effect on dry eyes

In this dry eye disease (DED) model, rabbit lacrimal glands are injected with Concanavalin A (ConA) a mitogen which triggers an immune response, causing glandular damage and leading to aqueous-deficient DED. The resulting tear fluid loss induces pathological changes to the cornea and conjunctival, including reduced corneal sensory nerve endings. Animals exhibit ocular changes which persist for one to 2 weeks. To establish a chronic DED model, repeated ConA injections are administered every 7–14 days.

That rabbits with an acute form aqueous-deficient DED show suppressed CS may seem paradoxical, given the short duration of their DED. However, such rapid changes in CS has been shown in other animal models (it cannot be determined in humans, as the time of initiation of DED is unknown). For example, it was recently shown in mice that acute desiccating stress reduces within 3 days intraepithelial corneal nerve density, CS, and apical extension of the intraepithelial nerve terminals (Stepp et al., 2018). The suppressed baseline CS score mirrors the human DED (Bourcier et al., 2005; Belmonte et al., 2017; Dien et al., 2015) although most human cases of dry eye disease is evaporative in contrast to our rabbit model. Whether PS has similar effects on other forms of dry eye will have to await further studies.

PS restored CS in NZW rabbits with aqueous-deficient dry eyes, which had suppressed baseline CS scores. PS was the only compound producing this effect; notably, PS had the exact opposite effect on normal eyes (as discussed below). Cyclosporine and lifitegrast and a representative brand of artificial tears failed to change CS in either dry or normal eyes. Of interest, cyclosporine has been reported to improve CS in patients with DED (Toker and Asfuroglu, 2010) and also following cataract surgery (Hamada et al., 2016). The ocular analgesic NSAIDs ketorolac and bromfenac suppressed the CS of dry eyes as well as normal eyes.

The effect of PS on CS in rabbits with dry eyes was essentially immediate and reversible, disappearing within a few hours. This observation indicates a direct effect of PS on the corneal nerves. Even though the inflammation of dry eye is thought to affect ocular nerves, the rapid onset and reversibility of PS’s effects suggest but do not prove that a non-anti-inflammatory mechanism may be part of its effect on CS.

Two factors significantly affected the efficacy of PS, its formulation and concentration. The hydrogel formulation was the most efficacious. CS was normalized for 27 h, an effect remarkably stable for such an extended time period, making once a day dosing a possibility. Previous pharmacokinetic studies indicate that PS administered in eye drop form is present in the cornea for several hours. However, in the current study, the effect of PS on CS persists for a much longer period when compared to the measured presence of PS suggesting other downstream changes affecting CS. Two solutions and one emulsion formulation restored CS, but their effect was of a much shorter duration, normalizing it for about 1 h. Interestingly, the emulsion formulation (F6) that produced a strong effect in dry eyes was ineffective in normal eyes. The dose dependence of PS’s effect, clear from our results, is remarkable for its sharp transition towards efficacy. This transition occurs between 0.1% and 0.2%, with a plateau above 0.2%; an 8-fold higher dose failed to enhance PS’s efficacy.

The restoration of CS in dry eyes by PS may be relevant to the treatment of DED, since the majority, if not all, of its symptoms are mediated through the nerves of the ocular surface (Belmonte et al., 2017). Others have shown that DED in both humans and animal models is associated with changes in the corneal nerve anatomy, distribution and density of nerve endings. The cornea also has three different classes of sensory afferents responding to different stimuli. For example, mechanical stimuli activates both mechanosensory and polymodal nociceptors. Painful stimuli active polymodal nociceptors and cold stimuli activate “cold” fibers (Belmonte et al., 1999). In our rabbit model we use a Cochet-Bonner esthesiometer to measure corneal sensitivity in both dry eye and normal animals. Using a blink response and eye retraction as a “positive response” to determine corneal sensitivity means that of the remaining corneal nociceptors present in dry eye animals, we assume activation of mechanosensory and polymodal receptors which in turn activate the blink reflex. The loss of corneal sensitivity in dry eye would seem to be an effect of a decrease in nociceptor density. The rapid increase in CS after the administration of PS we speculate would seem to be associated with an increase in the activity of the remaining ‘mechanosensory/polymodal’ nociceptors verses a sudden increase in the density of the corneal nerve fibers. Confirmation of our hypothesis would require electrophysiologic, immunohistochemical and molecular studies.

Topically administered, PS is very efficacious and appears safe in animal models of DED. Its effect on CS, adding immediate symptomatic relief to its anti-inflammatory effect could be therapeutically important in DED (provided that our results are translatable to humans).

### 4.2 Effect on normal eyes

PS suppressed CS in normal eyes, the opposite of its effect in dry eyes. Nevertheless, the two effects shared similar attributes, being in both strong, immediate, and dependent on drug formulation and dose. The formulation dependence was best shown by the distinct formulations F1, F5 and F7, which, despite having the same drug concentration (3.5%) gave sharply different results. Similar to dry eyes, hydrogel was the most successful, but its effect was considerably shorter (<2 h vs. 27 h). There is no ready explanation for this striking difference; early data show no difference in the ocular biodistribution of PS.

Unexpectedly, the gelan gum-based formulation, an *in situ* gelling system (Destruel et al., 2017; Osmalek et al., 2014), was not efficacious. Although several factors may have contributed to the better result of the Carbopol-based gel (F8), the viscosity-enhancing properties of Carbopol may have played a significant role. Of the solution formulations, F3 (18% HP-β-CD, pH 6.0) performed best. Formulations with more HP-β-CD (36% and 66%) were also efficacious, but their higher viscosity and possible regulatory barriers may make them impractical. The F6 solution formulation with PS 0.2% was also excellent. The emulsion and nanoparticle formulations had borderline effects.

The dose dependence of PS’s efficacy, evident in all studies, is most clearly demonstrated in the study using four drug concentrations of PS in the F4 solution formulation. Here too, the transition to efficacy was sharp and reached abruptly a plateau at 0.2% PS.

In the HP-β-CD-based solution formulations, the efficacy of PS was directly dependent on pH. The optimal pH was 6.2. Cyclodextrins, cyclic oligomers of glucose, can form water-soluble inclusion complexes with small molecules or portions of large compounds (Sharma and Baldi, 2016; Davis and Brewster, 2004). Their cup architecture, aqueous solubility due to their hydrophilic exterior, and the hydrophobic micro-environment of the apolar interior of the cup allows them to encapsulate and deliver lipophilic molecules like PS. There is a dynamic equilibrium between the cyclodextrin, free drug molecule and their inclusion complex. Studies with drugs similar to PS, e.g., trifluoperazine (Lutka and Golda, 2006) and derivatives of anthranilic acid like the NSAID mefenamic acid (Domanska et al., 2011), have demonstrated that pH greatly affects the stability constant of the drug-HP-β-CD inclusion complexes. Thus, it is entirely likely that a similar mechanism operated in our case, explaining the impressive pH-dependence of our cyclodextrin-based formulation.

The effect of PS on CS was confirmed in two strains of rabbits differing in their melanin content. This was important, as melanin can function as a molecular sink, sequestering significant amounts of small molecule drugs, occasionally reducing their efficacy (Rimpela et al., 2018). In our study, the pigmented Dutch-belted and the albino NZW rabbits responded similarly to PS, validating the latter. We also note a difference of the response to vehicle when we measured the CS in these two groups.

Evaluation of 12 additional compounds provided an interesting comparison to PS. First, all six compounds structurally related to PS (sulindac; the sulindac derivative, Q-922; the derivative of ibuprofen; and two derivatives of aspirin) failed to suppress CS. Similarly, cyclosporine, lifitegrast and the artificial tears did not affect CS in normal eyes (as in dry eyes). The lack of an effect of cyclosporine or lifitegrast on CS in these studies may have to do with their delayed therapeutic onset. Patients with DED do not usually experience relief of their symptoms for several weeks. Lidocaine and the analgesic and anti-inflammatory NSAIDs ketorolac and bromfenac (Kim et al., 2010) suppressed CS. These two NSAIDs are known to suppress CS (Singer et al., 2015; Aragona and Di Pietro, 2007), presumably by suppressing the corneal levels of prostaglandin E_2_ (Bucci and Waterbury, 2011). A significant limitation of these compounds, aside from rather intense instillation discomfort, is their association with corneal melt, a potentially devastating side effect (Rigas et al., 2019). Like ketorolac and bromfenac, PS suppressed CS in rabbits with normal eyes. However, that is where the similarity ends, since PS, not being a NSAID, does not suppress PG production in the cornea; a side-by-side comparison of PS and ketorolac in rabbits has established this difference (Honkanen et al., 2018). Furthermore, PS suppresses the activity of matrix metalloproteinases (Honkanen et al., 2018), in contrast to ocular NSAIDs which cause corneal melt (Rigas et al., 2019). As we have argued, compounds like PS that preserve the cytoprotective ocular PGE_2_ and restore CS in rabbits with reactivation of a blink/tear reflex may have a distinct therapeutic advantage over conventional NSAIDs (Rigas et al., 2019).

### 4.3 Summary and conclusion

Overall, PS displays a remarkable effect on CS characterized by several pharmacological attributes: structural specificity; better efficacy than other therapeutically relevant compounds; a range of suitable formulations, one allowing the possibility of once-a-day dosing and another allowing efficacy calibration by pH adjustment; therapeutic flexibility based on its differential effects on normal and dry eyes; and the advantage of combining the restoration of CS with a therapeutic effect on DED.

The clear dissociation of the analgesic and anti-inflammatory effects may help provide an insight into the pathophysiology of DED. The stunning dichotomy between the response of normal and dry eyes indicates either some change in the nerves of DED that alters their response to PS or the *ab initio* presence in the cornea of two different nerve populations, each dominating the PS response in each context (normal vs. DED). In light of the loss of sensory receptors in DED, an alternate explanation for these “contrasting” results could be related to a direct effect of PS on the mechanosensory and polymodal nociceptors themselves. If we consider the measurement of CS by Cochet-Bonnet esthesiometer as a simple stimulus response, in the setting of dry eye disease, PS may increase the sensitivity of the remaining sensory nerve endings. If in normal eyes PS activates the same sensory corneal receptors, it may be possible that the sensory nerves are “overwhelmed” or depolarized beyond the threshold necessary to propagate a mechanosensory stimulus.

These unique contrasting effects of PS on normal and dry eyes suggest two potential clinical applications. First, as a topically effective treatment for ocular pain and inflammation in “normal eyes”, such as, for example, post cataract surgery or for local injuries, and second, in the treatment of DED. Based on these considerations, it appears that PS merits further evaluation for its potential ocular therapeutic applications.

## Data Availability

The raw data supporting the conclusions of this article will be made available by the authors, without undue reservation.
