# Supplementary material for: Phosphosulindac (OXT-328) restores the suppressed corneal sensitivity in rabbits with dry eye disease: therapeutic implications
Source: Front Drug Deliv. 2026 Jun 3;6:1778440. doi: 10.3389/fddev.2026.1778440 (PMC13272426; doi:10.3389/fddev.2026.1778440)
Supplement: Supplementary file 1 [file Supplementaryfile1.pdf]

Supplementary Table (Table S1)

Table S1. Detailed composition of phosphosulindac (PS) ocular formulations

Formulation #	PS, %	Excipients	pH
F1 *solution*	3.5	66% HP-β-CD, 1% Tween 80	4.0
F2 *solution*	1.0	36% HP-β-CD, 1% Tween 80	4.0
F3 *solution*	0.5	18% HP-β-CD, 1% Tween 80	4.0, 6.0, 6.7, 7.4, 8.0
F4 *solution*	0.05, 0.1, 0.2 or 1.6	10% HP- β-CD, 4% Tween 80, 2.5% Vitamin E TPGS, 1.4% PVA, 0.001% , polyquaternium-1	7.4
F5 *solution*	3.5	16% Vitamin E TPGS, 3.18% mannitol, 1.2% boric acid, 0.005% polyquaternium-1	6.7
F6 *emulsion*	1.0 or 2.0	5% Propylene glycol, 10% Mineral oil, 4% Tween 60, 4% Tween 80, 10% HP- β-CD	6.1
F7 *nanoparticles*	3.5	20% mPEG-PLA in PBS	7.1
F8 *hydrogel*	0.2 or 0.6	10% HP- β-CD, 4% Tween 80, 3% mannitol, 1.25% Vitamin E TPGS, 0.8% PVA, 0.45% Carbopol 980, 0.001% polyquaternium-1	6.4
F9 *in situ gel*	3.0	0.5% gellan gum, 5% Vitamin E TPGS, 10% HP- β-CD	6.7

PVA: poly (vinyl alcohol); Vitamin E TPGS: D-α-tocopheryl polyethylene glycol 1000 succinate; mPEG-PLA: methoxy poly (ethylene glycol)-poly (lactide).
